# Ténosynovite tuberculeuse des extenseurs de la main : localisation rare de la tuberculose

**DOI:** 10.11604/pamj.2014.17.166.3568

**Published:** 2014-03-06

**Authors:** Faten Frikha, Zouhir Bahloul

**Affiliations:** 1 Service de Médecine interne, CHU Hédi Chaker 3029, Sfax, Tunisie

**Keywords:** Ténosynovite, tuberculose, main, tenosynovitis, tuberculosis, hand

## Image en medicine

La ténosynovite tuberculeuse de la main est une manifestation rare de la tuberculose et représente 5% des tuberculoses ostéoarticulaires et prédomine au poignet et à la face palmaire de la main. Homme âgé de 52 ans, aux antécédents de tuberculose pulmonaire, était hospitalisé pour une tuméfaction indolore du tendon extenseur du 3ème doigt de la main gauche et une douleur de l'articulation MCP du 3ème doigt droit dans un contexte apyrétique. L'examen notait une tuméfaction non inflammatoire peu douloureuse rénitente et crépitante en regard du tendon extenseur du doigt gauche faisant 3 cm de diamètre. Le reste de l'examen était normal. A la biologie, la vitesse de sédimentation était à 14 mm à la première heure et la C-réactive protéine à 6 mg/l. Il existait une hyperleucocytose à 11340 éléments/mm3. L'intradermoréaction à la tuberculine était phlycténulaire à 25 mm. La recherche de bacille de Koch dans les crachats et les urines était négative. L’échographie montrait un épaississement synovial avec épanchement en regard de l'articulation MCP des 3èmes doigts droit et gauche. L'IRM mettait en évidence en regard de l'articulation MCP gauche un épaississement synovial de réhaussement hétérogène ainsi que des images de collections liquidiennes péri-articulaires à paroi réhaussée dont la plus volumineuse mesurait 31X17X10 mm. Un traitement chirurgical était réalisé avec une synovectomie totale, un lavage articulaire et une immobilisation articulaire par une broche croisée. L’étude histologique montrait un granulome épithéliogigantocellulaire avec nécrose caséeuse. Le patient était traitée par antituberculeux pendant 12 mois. L’évolution était bonne.

**Figure 1:**
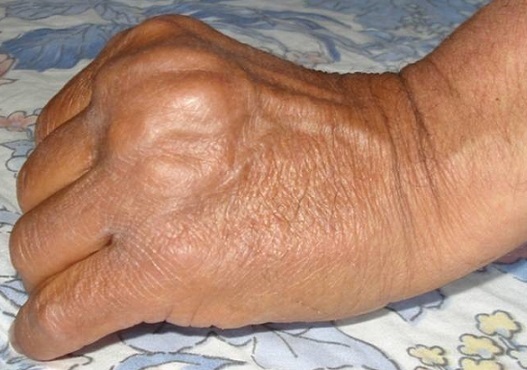
Tuméfaction non inflammatoire peu douloureuse rénitente et crépitante en regard du tendon extenseur du 3ème doigt gauche faisant 3 cm de diamètre

